# Supplementary material for: A novel simian adenovirus-vectored COVID-19 vaccine elicits effective mucosal and systemic immunity in mice by intranasal and intramuscular vaccination regimens
Source: Microbiol Spectr. 2023 Oct 25;11(6):e01794-23. doi: 10.1128/spectrum.01794-23 (PMC10715068; doi:10.1128/spectrum.01794-23)
Supplement: Fig. S1-S6 — Supplemental material. [file spectrum.01794-23-s0001.pdf]

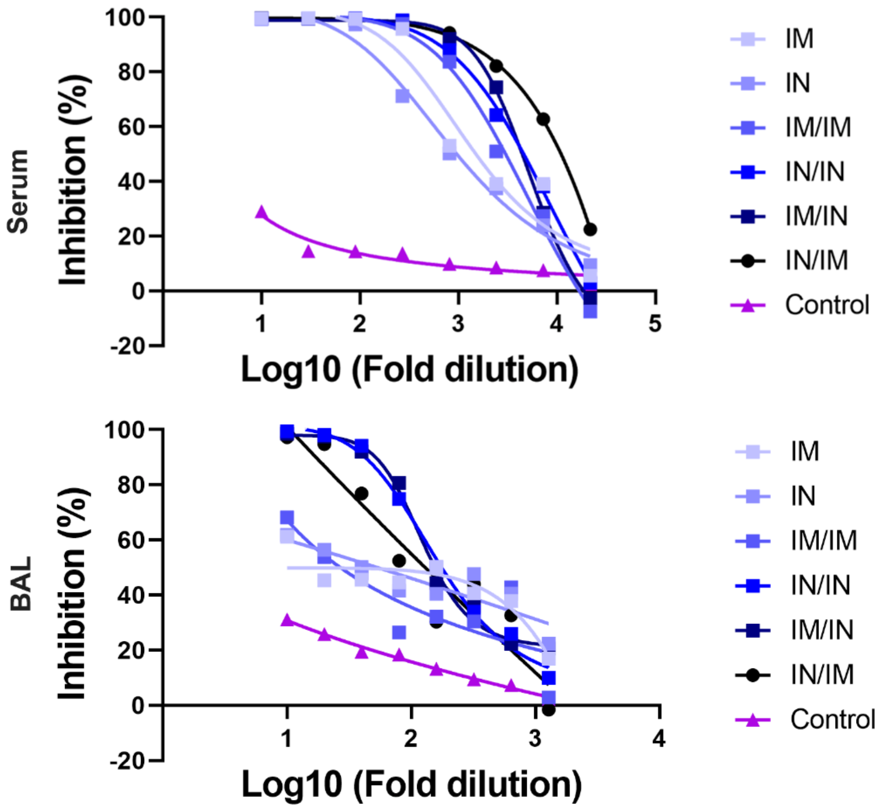

Figure S1. Titration of serum and BAL NAb in different immunization schemes against pseudovirus of SARS-CoV-2 by pVNT. Titration of NAb titers from serum and BAL samples were carried out by pVNT with pseudovirus. Serum and BAL samples were obtained at four weeks after a single or prime-boost immunizations.

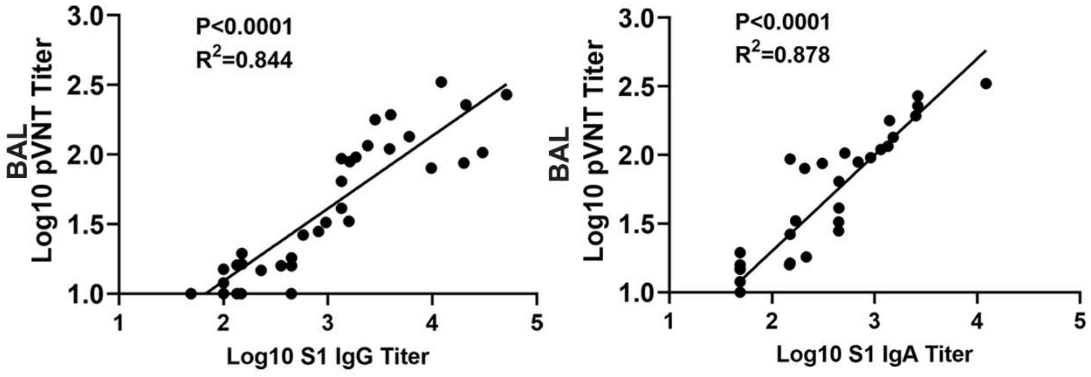

Figure S2. Correlation between S1-BAb and NAb titers. Correlation between IgG or IgA S1-BAb and NAb titers in BAL samples to wild-type was compared from all vaccination regimens using Pearson's correlation coefficients, respectively.

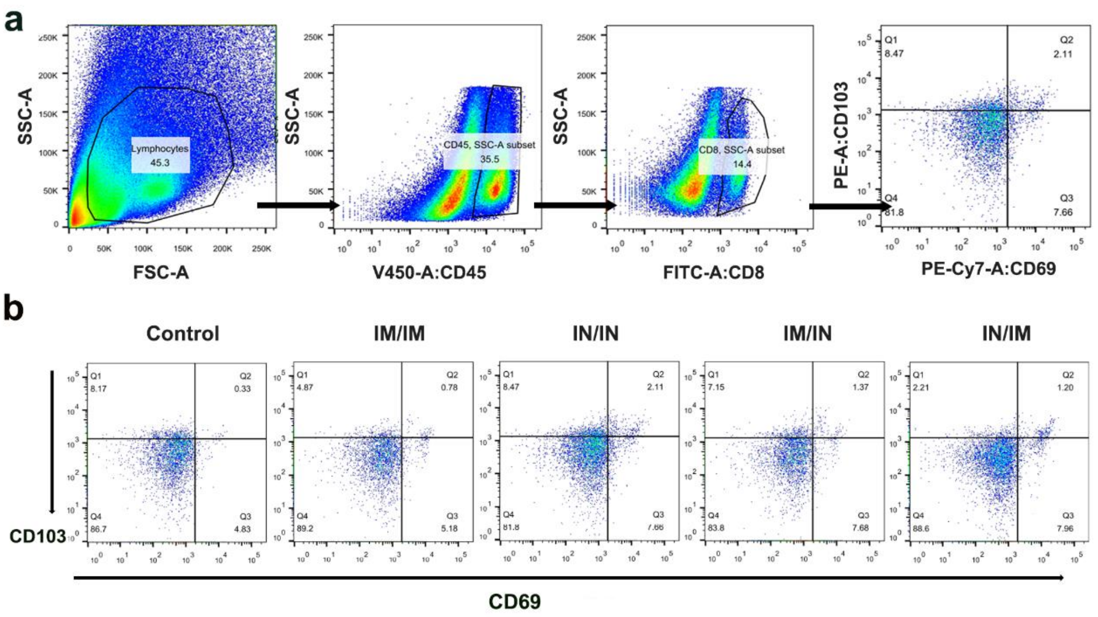

Figure S3. Determination of resident memory T cells (TRM cells) expressing marker CD103 and CD69 in lung of mice from four prime-boost groups. (a) Flow cytometry data gating strategy. All events were first depicted using a combination of forward scatter area (FSC-A) and side scatter area (SSC-A). All leukocyte subsets were distinguished by first gating $CD45^+$ cells. Q2 ($CD69^+CD103^+$) area represented TRM cells proportion. **(b)** TRM cells proportion from four prime-boost groups were shown.

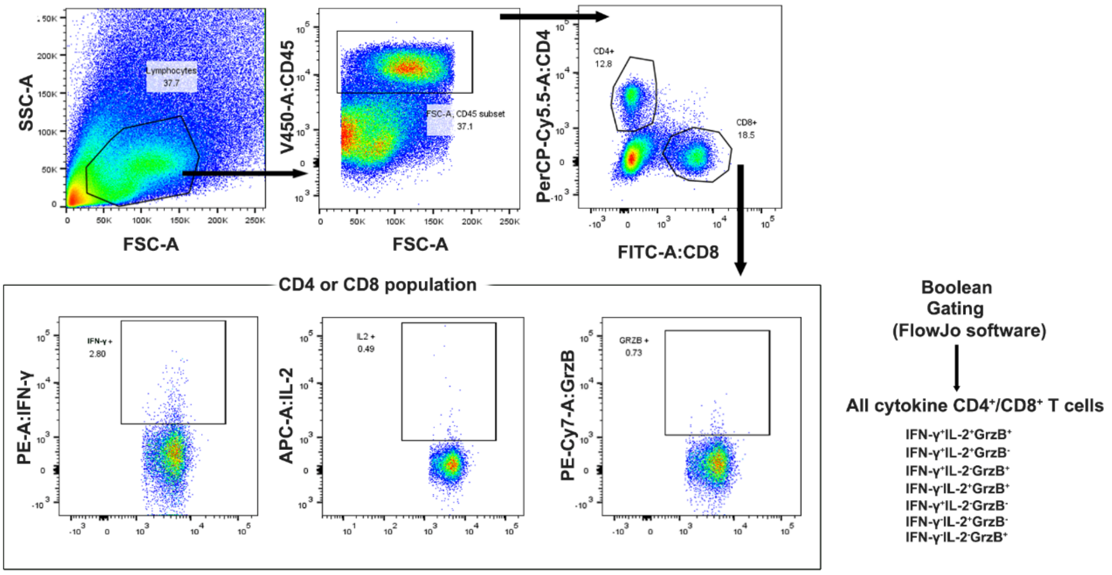

Figure S4. Flow cytometry gating strategy for T cell analysis. Gating strategy was used to analyze CD4 T cells, CD8 T cells, cytokine^{+} CD4 and CD8 T cells in spleen and lung. All leukocyte subsets were distinguished by first gating CD45^{+} cells. CD4^{+} and CD8^{+} gating was applied and analyzed for cytokine^{+} (IFN-γ, IL-2, and GrzB) expression. To determine the frequency of all cytokine^{+} CD4 and CD8 T cells subsets, boolean gating was applied to the gated IFN-γ^{+} cells, IL-2^{+} cells, GrzB^{+} cells.

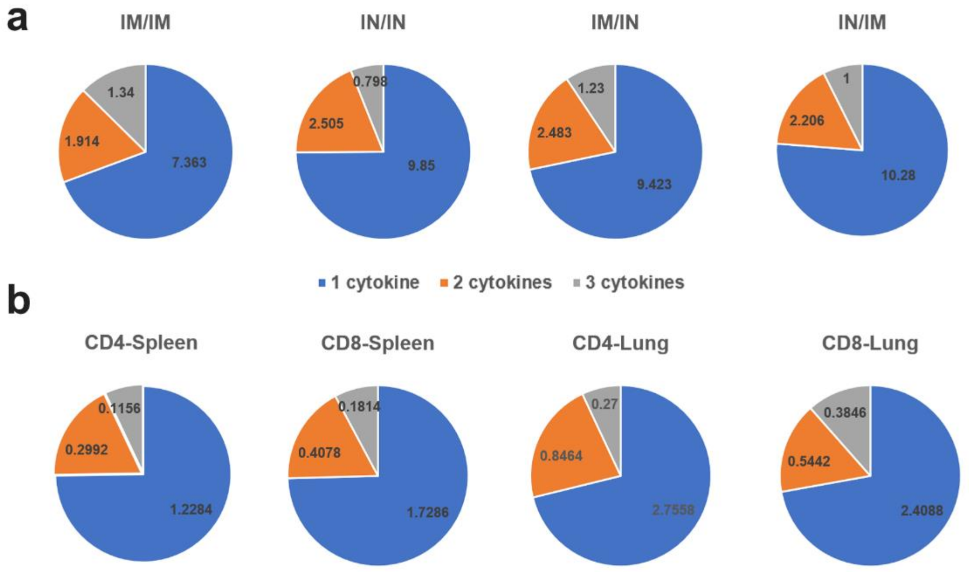

Figure S5. The proportion of functional T cells from different prime-boost groups and different tissues. **(a)** Cytokine-secreting (IFN-γ^+, GrzB$^+$, or IL-2$^+$) T cell expression only one or multiple cytokines from four prime-boost vaccinations. **(b)** The proportion of cytokine$^+$ CD8$^+$ and CD4$^+$ spleen and lung T cells expressing 1, 2, or 3 cytokines.

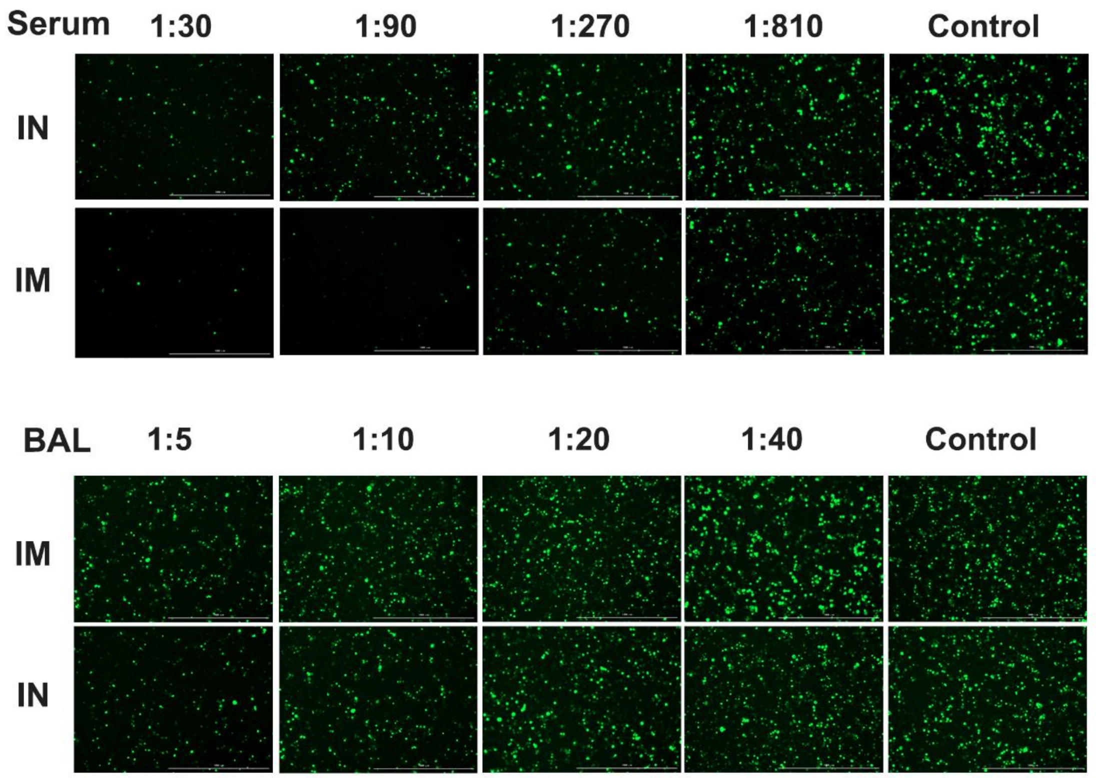

Figure S6. AdNAb to Sad23L vectors were measured from serum and BAL samples in mice 4 weeks post prime IM or IN vaccination. The serum samples were serially diluted in 1:3 times starting from 1:30, and BAL was serially diluted in 1:2 times starting from 1:5.
